# Mesoporous Copper‐Based Metal‐Organic Framework Flakes as a Promising Platform for Electrosynthesis of Ethylene from Carbon Dioxide

**DOI:** 10.1002/smsc.70323

**Published:** 2026-06-11

**Authors:** Mitra Bagheri, Mirtha A. O. Lourenço, Micaela Castellino, Silvia Nappini, Felicia Di Costola, Julien K. Dangbegnon, Elena Magnano, Luís Mafra, Fabrizio Pirri, Adriano Sacco, Juqin Zeng

**Affiliations:** ^1^ Istituto Italiano di Tecnologia – IIT Center for Sustainable Future Technologies (CSFT) Turin Italy; ^2^ Department of Applied Science and Technology (DISAT) Politecnico di Torino Turin Italy; ^3^ CICECO – Aveiro Institute of Materials Department of Chemistry University of Aveiro Aveiro Portugal; ^4^ CNR ‐ Istituto Officina dei Materiali (IOM) Trieste Italy

**Keywords:** carbon dioxide electroreduction, copper, ethylene, metal‐organic framework, operando x‐ray absorption spectroscopy

## Abstract

Developing practical electrocatalysts for carbon dioxide electroreduction (CO_2_RR) requires a synergy of sustainable synthesis and high performance. Here, we report a one‐pot green synthesis of a flake‐like, mesoporous copper‐based metal‐organic framework (Cu‐MOF) optimized for CO_2_ conversion. Spectroscopic analysis confirms a coordination environment of Cu^2+^ centers tetragonally bonded to nitrogen atoms, characteristic of zeolitic imidazolate frameworks (ZIFs). This flake‐like architecture and mesoporosity facilitate rapid mass transport and ensure maximum site accessibility. By maintaining optimally spaced Cu centers, the framework modulates the surface coverage of reaction intermediates, effectively promoting C–C dimerization toward multicarbon products. Operating in a zero‐gap cell, the Cu‐MOF achieves a Faradaic efficiency of 51.6% for ethylene at an industrially relevant current density of 200 mA cm^–2^. Furthermore in‐situ X‐ray absorption spectroscopy (XAS) reveals a reversible restructuring into metallic Cu active species with negligible dissolution, demonstrating the robust potential of this green‐synthesized catalyst for scalable CO_2_ electrolysis.

## Graphical abstract

We report a one‐pot green synthesis of a flake‐like, mesoporous Cu‐based metal‐organic framework for CO_2_ conversion. Its structure enhances mass transport and active‐site accessibility, promoting C–C coupling. In a zero‐gap cell, it achieves 51.6% C_2_H_4_ selectivity at 200 mA cm^–2^. In situ X‐ray absorption spectroscopy shows reversible formation of metallic Cu active sites with negligible dissolution, highlighting durability under reaction conditions.

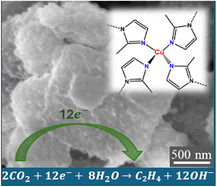

## 1 Introduction

The escalating global energy demand and the environmental urgency of greenhouse gas mitigation necessitate the transition toward a carbon‐neutral economy [1, 2]. Electrocatalytic CO_2_ reduction (CO_2_RR) represents a compelling route to transform waste CO_2_ into value‐added fuels and feedstocks [3, 4, 5, 6, 7]. Among current electrocatalysts, copper (Cu)‐based materials have emerged as leading electrocatalysts for CO_2_RR due to their unique ability to produce a wide range of C_1_ and C_2+_ products, including hydrocarbons and alcohols [8, 9, 10, 11, 12]. Current research has extensively explored Cu‐based architectures to tune product distribution. For instance, supporting Cu on functionalized multiwalled carbon nanotubes [13] or alloying with metals like Sn and Sb [12, 14, 15, 16, 17] has proven effective for directing selectivity toward C_1_ products like CO and formate. However, the unique ability of Cu to catalyze C–C coupling has shifted the focus toward optimizing C_2+_ production. Strategies such as alloying with secondary metals, including Re, Ag, Al, and Zn [18, 19, 20, 21] have demonstrated significant enhancements in ethylene and ethanol selectivity. Beyond compositional tuning, precise control over physical and electronic parameters, such as particle size [22], grain boundary density [23], facet exposure [24], and oxidation state modulation [25] remains pivotal in promoting the formation of high‐value multicarbon products. Among current catalyst platforms, copper‐based metal‐organic frameworks (Cu‐MOFs) have emerged as premier candidates for CO_2_RR due to their high surface areas and precisely defined active sites [26]. Unlike traditional bulk metals, the periodic assembly and modularity of the MOF architecture allow for the precise engineering of mutimetallic sites at the atomic level [27]. This structural precision enables the systematic modulation of reaction pathways by regulating the adsorption energy and spatial geometry of reaction intermediates [27]. For instance, recent categorizations highlight that adjacent multicopper centers with similar coordination environments are specifically conducive to C−C coupling for ethylene (C_2_H_4_) generation. Beyond basic composition, tailoring the first coordination shell of metal centers has emerged as a powerful strategy to optimize activity [28]. By modulating ligand side groups in MOF precursors, researchers have developed catalysts with hybrid coordination, that shift the d‐band center toward the Fermi level, significantly lowering the free energy barrier for the formation of key intermediates like *COOH and enabling industrial‐scale current densities [28]. Yao et al. reported a core–shell MOF‐derived Cu catalyst (Cu@Cu_*x*_O) via a multistep solvothermal synthesis of MOF using hazardous solvents followed by restructuring, achieving a ∼51% faradaic efficiency for C_2_H_4_ at −1.58 V versus the reversible hydrogen electrode (RHE) and a C_2_H_4_ partial current density of ∼150  mA cm^−2^ in a flow cell [29]. Similarly, Tan et al. fabricated a Cu_2_O@Cu‐MOF core–shell catalyst through a multistep, solvent‐assisted synthesis involving aqueous chemical reduction to form Cu_2_O spheres, followed by in situ chemical etching and coordination growth of a Cu‐BTC MOF shell using organic solvents; the catalyst was applied to CO_2_ electroreduction in an H‐cell and achieved a C_2_H_4_ faradaic efficiency of ∼16% at −1.71 V versus RHE under relatively low current densities (<20 mA cm^−2^) [30]. While Zhao et al. reported a Cu(111)@Cu‐THQ tandem catalyst prepared through a multistep process involving solution‐based synthesis of a conductive Cu‐THQ MOF, electrode casting with organic solvents, and in situ electrochemical reduction to generate Cu(111) nanoparticles; when evaluated for CO_2_ electroreduction in an H‐cell, the catalyst achieved a C_2_H_4_ selectivity of ∼44% at −1.2  V versus RHE with current densities of ∼8–15  mA cm^−2^ [31]. Besides being catalysts, the MOF‐based membranes also allow for in‐situ enrichment of CO_2_, facilitating the production of high‐purity, electrolyte‐free fuels directly from dilute streams [31, 32]. However, most reported Cu‐MOF materials still suffer from complex synthesis, reliance on toxic organic solvents, and limited selectivity under industrially relevant conditions.

To address these systemic gaps, we introduce a one‐pot green, steam‐assisted hydrothermal synthesis of a Cu‐MOF that eliminates toxic organic solvents and drastically streamlines the fabrication process. Beyond molecular design, we demonstrate that the resulting mesoporous, flake‐like morphology is a critical architectural feature; it simultaneously enhances mass transport, maximizes active site exposure, and regulates intermediate coverage. Our design leverages 2‐methylimidazole linkers to stabilize atomically dispersed Cu centers, a configuration specifically engineered to promote the C–C coupling kinetics essential for C_2+_ production. Following comprehensive structural elucidation, the Cu‐MOF was integrated into gas diffusion electrode (GDE) architectures. Performance was evaluated in both the flow‐cell and zero‐gap cell, where the catalyst delivers high C_2_H_4_ selectivity at industrially relevant reaction rates.

## 2 Results and Discussion

### 2.1 Physicochemical Properties of Cu‐MOF

**FIGURE 1:**
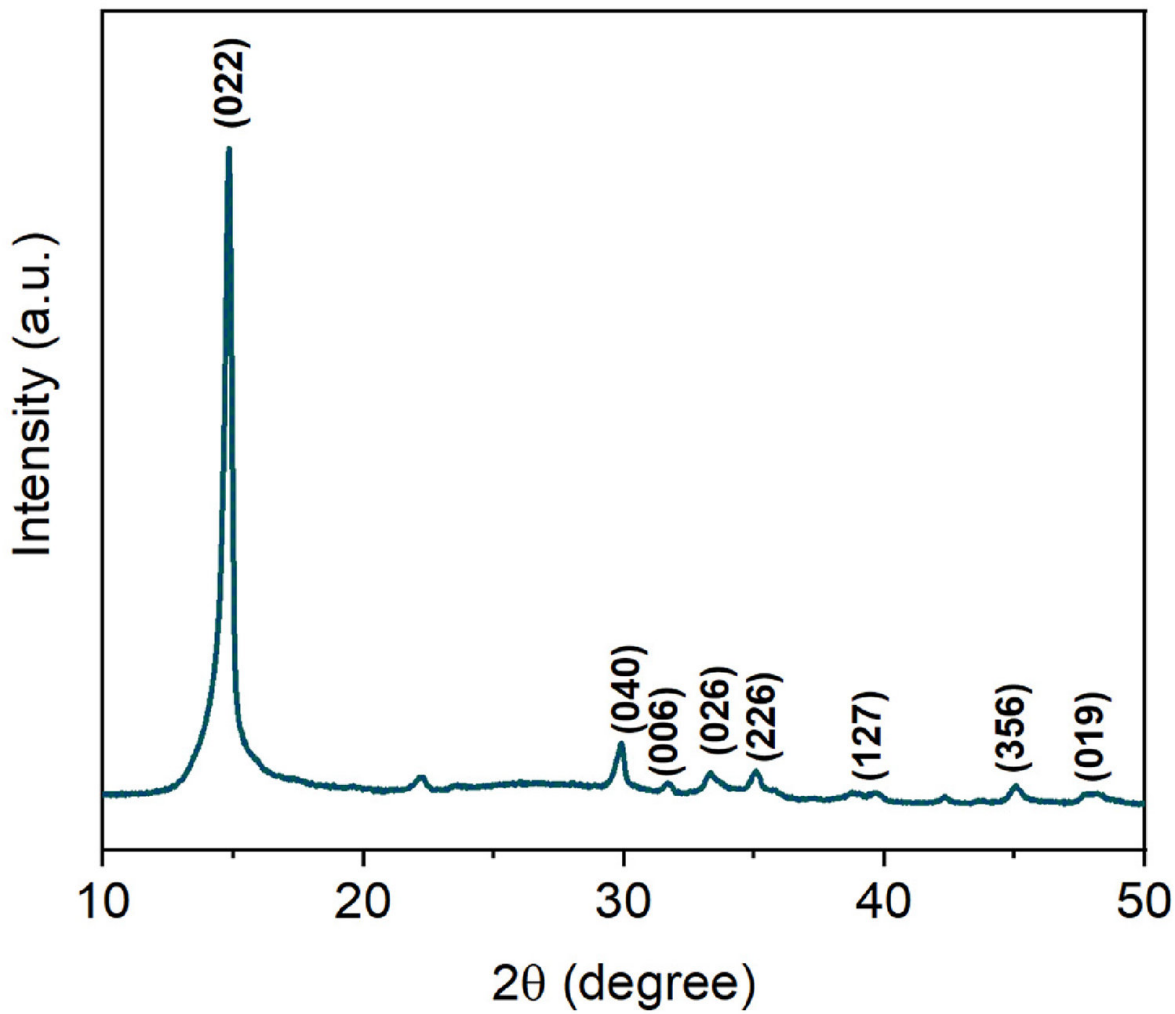
XRD pattern of Cu‐MOF.

The X‐ray powder diffraction (XRD) pattern of the Cu‐MOF exhibits a series of sharp diffraction peaks, indicating the successful formation of the Cu‐MOF with a well‐defined crystal structure, as shown in Figure 1. The diffraction peaks observed at 2*θ* = 14.6°, 29.8°, 31.5°, 33.3°, 35.7°, 38.8°, 44.9°, and 48.0° are attributed to the (022), (040), (006), (026), (226), (127), (356), and (019) diffraction planes, respectively, suggesting that the synthesized Cu‐MOF shares a similar crystal structure to those previously reported in literature [33]. No additional impurity peaks are observed, implying the high purity of the final sample.

**FIGURE 2:**
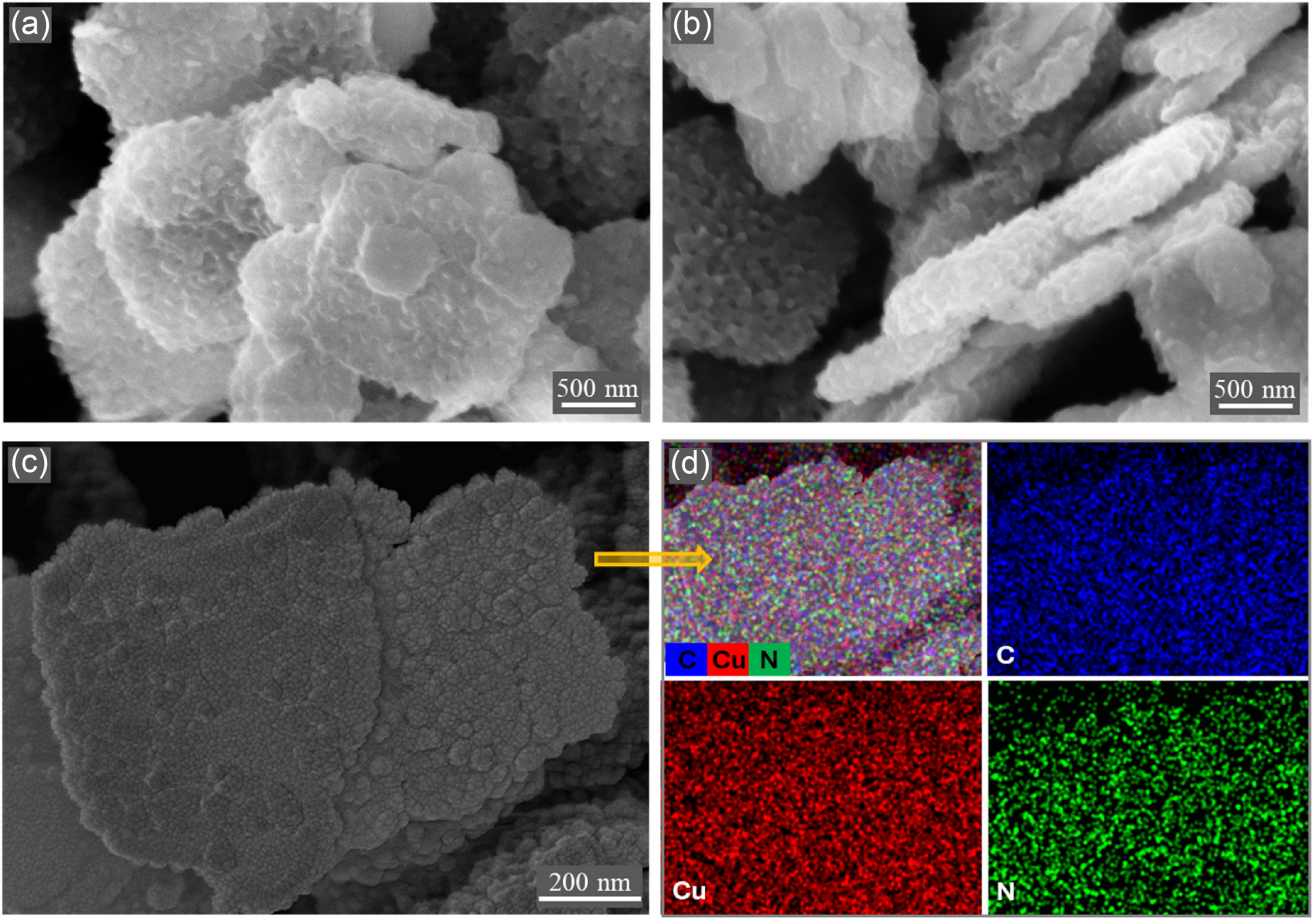
FESEM images of Cu‐MOF: (a) top view of the particles and (b) lateral view of the particles. (c) SEM image of Cu‐MOF under EDS condition and (d) EDS mapping.

The scanning electron microscopy (SEM) micrographs (Figure 2a,b) reveal densely packed grains forming irregular flake‐like nanostructures with thicknesses ranging from approximately 50–80 nm and dimensions vary between 200 and 1200 nm. The surfaces of these flakes exhibit notable roughness, marked by numerous protrusions and indentations. This rough texture, which is likely due to the presence of pores, a characteristic feature of MOF structures. Figure 2c,d shows energy‐dispersive X‐ray spectroscopy (EDS) mapping on the Cu‐MOF flakes, evidencing the uniform distribution of Cu, C, and N.

**FIGURE 3:**
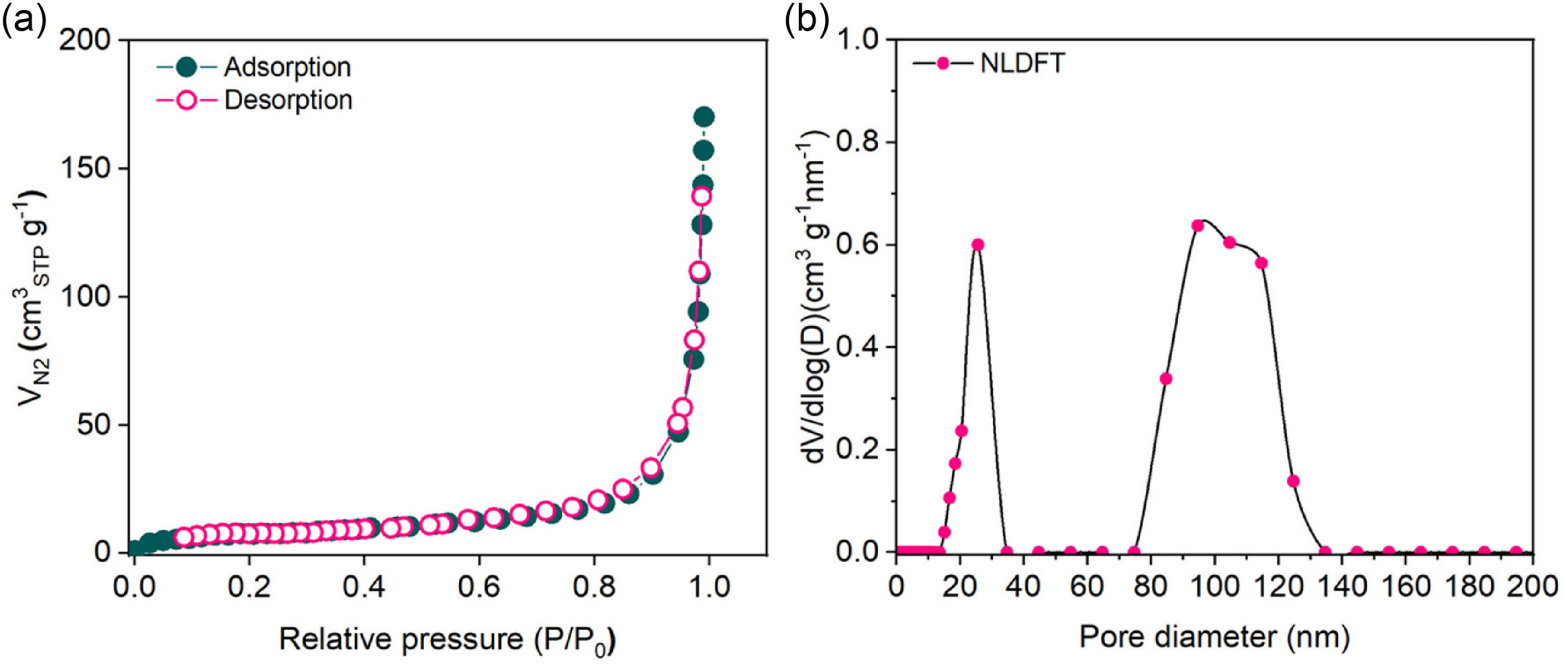
(a) –196 N_2_ adsorption–desorption isotherms (closed symbols correspond to the adsorption process, whereas empty symbols are associated with the desorption process) and (b) NLDFT pore size distributions of Cu‐MOF.

N_2_ adsorption–desorption measurements were performed to investigate the porosity of the Cu‐MOF. As illustrated in Figure 3a, the Cu‐MOF exhibits a typical type IV adsorption isotherm curve (according to IUPAC classification) [34], with a slight hysteresis loop at high relative pressures, indicative of a mesoporous structure with large mesopores [34]. The Brunauer–Emmett–Teller (BET) specific surface area (*S*_BET_) was measured to be 25.6 m^2^ g^–1^, with a total pore volume of 0.24 cm^3^ g^–1^. Pore size analysis using the Barrett–Joyner–Halenda (BJH) method revealed an average pore diameter of 19 nm, consistent with mesoporosity. Additionally, the nonlocal density functional theory (NLDFT) analysis, using graphitic carbon as the model material, indicated bimodal porosity with the most abundant pore sizes centered at approximately 26 and 100 nm (Figure 3b). These results are consistent with previously reported two‐dimensional Cu‐MOFs [33, 35, 36]. The mesopores are likely located within the flakes, while the macropores are attributed to the void among the flakes (Figure 2a,b).

Elemental analysis, specifically carbon, hydrogen, nitrogen, and sulfur (CHNS) analysis, was conducted to determine the elemental composition of the synthesized Cu‐MOF. The analysis revealed weight percentages of carbon (40.6%), hydrogen (4.6%), and nitrogen (24.6%). The Cu content in the Cu‐MOF was quantified by thermogravimetric analysis (TGA) (Figure S1) and inductively coupled plasma‐optical emission spectroscopy (ICP‐OES). Both results consistently indicate that the Cu‐MOF contains approximately 25–28 wt% Cu. The combination of element analysis, TGA, and ICP‐OES analyses confirm that C, H, N, and Cu are the main elements of the Cu‐MOF, with an estimated Cu‐to‐N ratio of 1:4, suggesting that each copper ion is coordinated by four nitrogen atoms, similar to the coordination environment of zeolitic imidazolate frameworks (ZIFs) [35].

Spectroscopic techniques, including Fourier transform infrared spectroscopy (FTIR) and Raman were further employed to study the chemical composition and structure of materials. Figure S2 presents a comparative analysis of the FTIR spectra of 2‐methylimidazole and the synthesized Cu‐MOF, revealing a significant spectral shift and the emergence of new bands, indicative of metal‐ligand coordination beyond the bands of ligand. The Raman spectrum (Figure S3) of the synthesized Cu‐MOF confirms its successful formation, featuring sharp and intense peaks at 690, 1023, 1183, 1462, and 2920 cm^–1^. These peaks correspond to various vibrational modes of the 2‐methylimidazole aromatic ring: C = C stretching (690 cm^−1^), C–H bending (1023 cm^−1^), C–N stretching (1183 cm^–1^) within the imidazole rings, methyl bending (1462 cm^–1^), and C–H stretching of the methyl groups (2920 cm^–1^) [35]. Vibrational modes associated with the Cu─N bonds within the MOF typically appear in the lower frequency region of the Raman spectrum. The peak observed at 293 cm^–1^ can be attributed to the Cu–N vibration, although these modes are often obscured by overlapping vibrational modes, making precise identification challenging.

**FIGURE 4:**
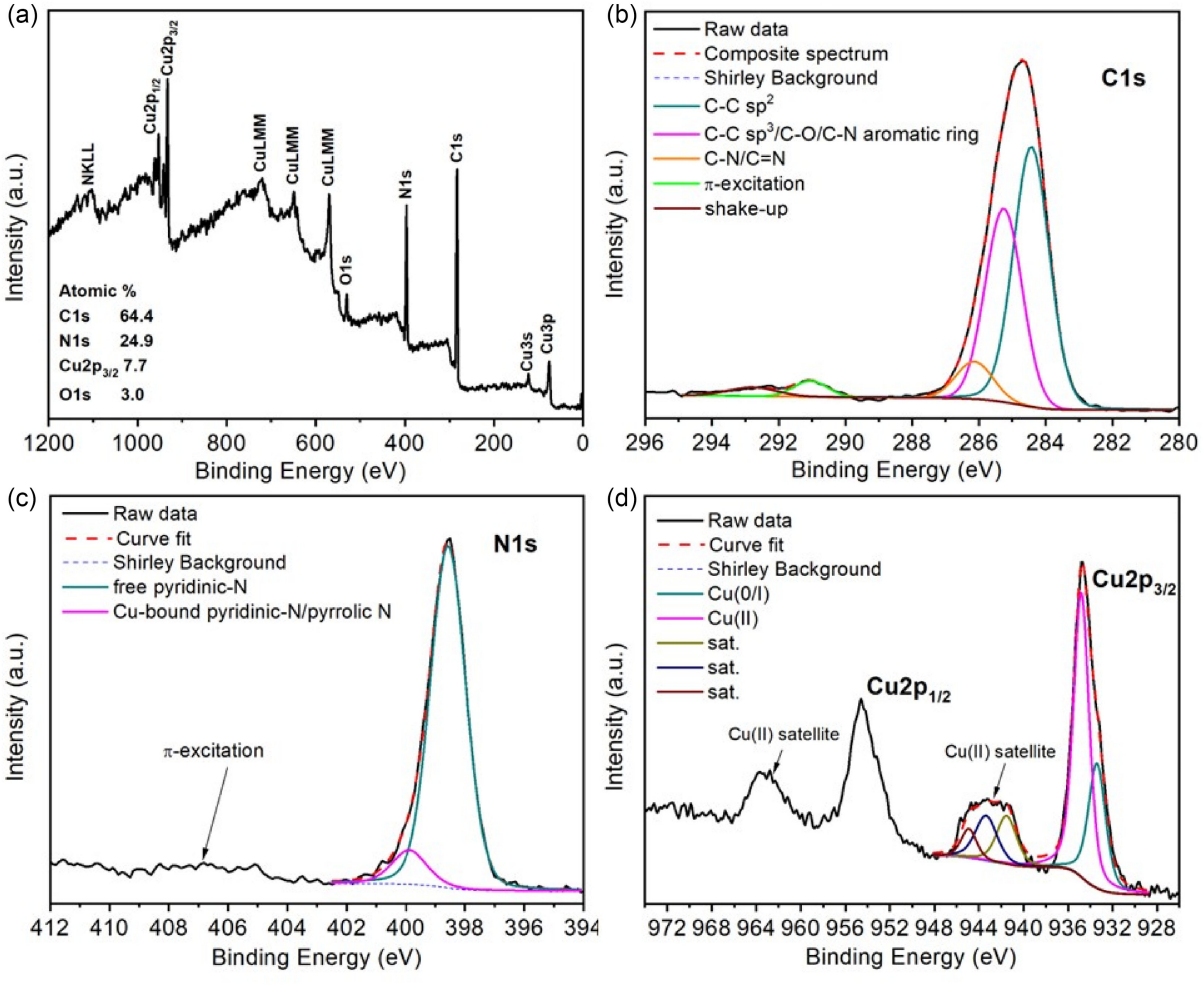
XPS survey (a) and HR curves of Cu‐MOF: (b) C 1s, (c) N 1s, and (d) Cu 2p regions.

X‐ray photoelectron spectroscopy (XPS) analysis was performed to obtain detailed information on the surface elemental composition and chemical environment for each element present. The survey spectrum (Figure 4a), displays characteristic peaks corresponding to C, O, N, and Cu, as expected. No additional elements were detected within the sensitivity of XPS (0.01 at.%). The relative atomic concentration was reported in the same figure, derived from quantitative analysis of the high‐resolution (HR) spectra (Figure 4b–d) providing improved accuracy, particularly for the noisy signal of the less abundant element (e.g., oxygen, not reported). The HR spectrum of the C 1s region (Figure 4b) shows a composite spectrum due to different contributions. The most intense component at 284.5 eV is attributed to the C─C bond in a sp^2^ configuration, while the second one at 285.3 eV is mainly due to C–C in a sp^3^ configuration and/or to C–O and C–N in an aromatic ring structure [37], not clearly distinguishable since the chemical shift regions overlap. An additional component at 286.1 eV is attributed to the CN/C═N bonds associated with amino configurations. Two additional components at higher binding energy values, 291.1 and 292.8 eV, respectively, are attributed to π excitation, common in aromatic ring molecules, and satellite shakeup, in sp^2^ carbon structures [38], which agree with the structure of 2‐methylimidazole precursors used for the synthesis of Cu‐MOFs. The N 1s spectrum (Figure 4c) exhibits a sharp peak centered at 398.6 eV with a small prominence around 399.9 eV, which are usually respectively ascribed to pyridinic‐N and to pyrrolic‐N, in agreement with the components already highlighted in the C 1s region [39]. A weak and broad satellite feature is also observed between 404 and 408 eV attributed to the π excitation, similar to that observed in the C 1s region. According to the literature reports, the free (uncoordinated) pyridine/aromatic nitrogen is predicted to be located in a region between ~398.3 and 399.5 eV, while the Cu‐coordinated nitrogen is shifted to a higher binding energy, generally in the range of 399.8–400.8 eV [40, 41, 42]. The shift is expected between +0.5 and +1.5 eV compared to the uncoordinated nitrogen. In the present case, the second weaker peak at 399.9 eV is shifted by 1.3 eV relative to the dominant pyridine N peak, which could therefore be attributed to a direct bond between the N and Cu atoms, which are predicted to be in the +2‐oxidation state. This shift originates from electron donation from nitrogen lone pair to Cu^2+^, which reduces electron density at nitrogen, raising its binding energy. To further substantiate this assignment, HR analysis of the Cu 2p region was performed (Figure 4d). The Cu 2p spectrum, comprising the 2p_3/2_ and 2p_1/2_ doublet, has a rather evident fingerprint, in the case where the +2‐oxidation state of Cu is present, since two easily recognizable satellite peaks are highlighted, at higher binding energies, compared to the two 2p_3/2_ and 2p_1/2_ peaks. In the present spectra, these satellite features are clearly observed (Figure 4d), providing direct evidence for the presence of Cu(II). However, the asymmetric shape of the Cu 2p_3/2_ peak suggests the presence of more than one oxidation state, due to the presence of a component with lower binding energy, around 933.4 eV. Since the components due to metallic copper, Cu(0), and the one due to the +1‐oxidation state, Cu(I), are at the same binding energy, it is not possible to distinguish between the two in this region. However, thanks to the method introduced by Biesinger et al. [43], it is possible to mathematically evaluate the relative percentage due to the +2‐oxidation state, with respect to 0 and +1, by a deconvolution of the Cu2p_3/2_ peak and its satellite, included in the region 940–950 eV. This fitting procedure yielded relative contributions of approximately 77% Cu(II) and 23% Cu(0/I). These results indicate that the majority of the Cu signal originates from the +2 oxidation state. The presence of this mix of oxidative states has already been reported in the literature, although no quantitative evaluation has been given, both for Cu‐MOF alone [44], and for Cu‐MOF composites with carbon matrices [45]. Therefore, for a direct Cu^2+^─N bond in an aromatic MOF matrix, we expect N 1s around 400 eV, a chemical shift in the C 1s region associated with C–N ~286 eV, and an additional Cu^2+^ fingerprint in Cu 2p region at ~934 eV with its correlated satellites. So, when we need confirmation of the presence of a Cu(II)─N bond (coordination of Cu^2+^ with N atoms in aromatic ligands such as imidazole, triazole, bipyridine, porphyrins, etc.), we need to check the N 1s region, since the chemical shift mainly occurs there. To further corroborate what was observed through XPS analysis and fill the gaps that emerged due to the overlaps in some regions of indistinguishable chemical shifts, it is necessary to use a complementary technique, such as X‐ray absorption spectroscopy (XAS).

**FIGURE 5:**
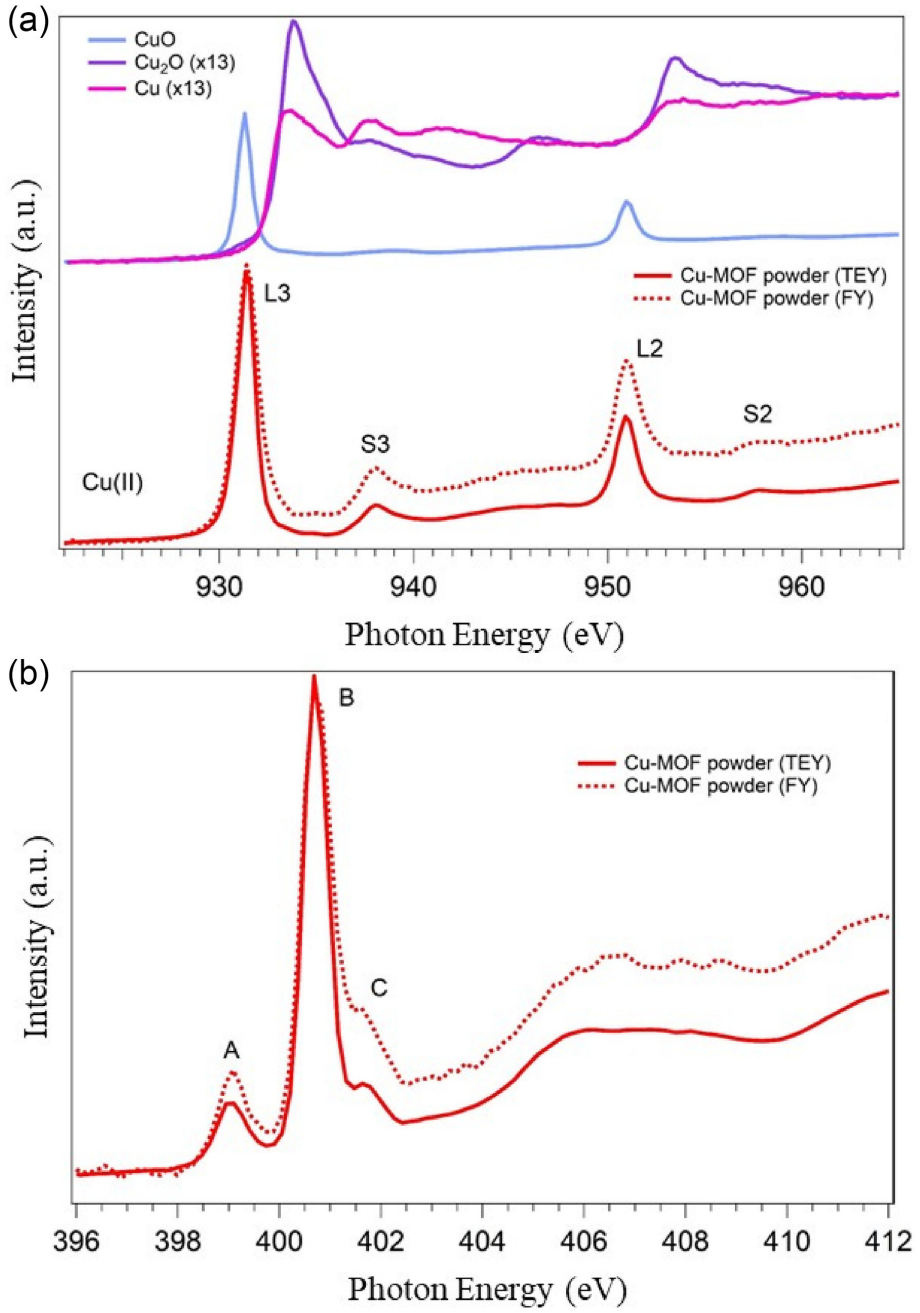
(a) Cu L‐edge soft‐XAS of Cu‐MOF powder recorded in TEY (red solid line) and FY (red dashed line), along with metallic Cu, Cu_2_O, and CuO reference spectra and (b) N K‐edge soft‐XAS spectra of Cu‐MOF powder recorded in TEY (red solid line) and FY (red dashed line).

Figure 5a presents the Cu L‐edge soft‐XAS spectra of the Cu‐MOF powder, compared with reference spectra of metallic Cu, Cu_2_O, and CuO. Measurements were conducted in both total electron yield (TEY) and fluorescence yield (FY) modes, allowing for depth‐resolved analysis from the near‐surface region (a few nanometers, TEY) to deeper layers (up to tens of micrometers, FY). The TEY and FY spectra display very similar overall line‐shapes, indicating that the surface and bulk regions share comparable composition. In both TEY and FY spectra, two main absorption features are observed at 931.5 eV (L_3_‐edge) and 951.1 eV (L_2_‐edge), corresponding to Cu 2p → 3d electronic transitions. These peaks are characteristic of Cu^2+^ species and closely resemble the CuO reference [46], though they are shifted by approximately +0.2  eV, indicating a slightly more oxidized or covalently bound Cu environment within the MOF framework. Notably, the Cu L_3_‐edge spectrum also displays a shoulder at ~938  eV (S3), which is not observed in the CuO reference. This feature indicates additional spectral complexity and is attributed to ligand‐to‐metal charge transfer (LMCT) transitions involving nitrogen‐based ligands within the Cu‐MOF. LMCT satellites, corresponding to 3d^9^ → 3d^10^ L excitations, have been widely reported in N‐coordinated Cu^2+^ systems, including NH_3_‐dosed Cu(I)‐MFU‐4l, Cu‐ZIF8, Cu‐ZIOS, and Cu‐ethylenediamine complexes [47, 48, 49]. These features are commonly linked to π‐backbonding interactions between Cu^2+^ centers and nitrogen donors. Similar LMCT‐related shoulders near 938.4 eV have been reported for Cu‐based MOFs such as HKUST‐1, where they are attributed to Cu–N or Cu–O charge transfer transitions [50]. A corresponding satellite is also observed at 957.8 eV in the Cu L_2_‐edge spectrum (S2), further supporting the presence of LMCT or multiplet effects involving interactions between ligand π orbitals and Cu d orbitals. These broad features, unlike sharp single‐electron transitions, arise from more delocalized or hybridized electronic states, consistent with LMCT involving N‐imidazole donors in the Cu^2+^ coordination environment [51, 52, 53, 54].

Although direct Cu L‐edge soft XAS studies on Cu‐methyl‐imidazole MOFs are scarce, the observed spectral features align well with known Cu–N coordination environments in imidazole‐based systems. Several density functional theory (DFT) and extended X‐ray absorption fine structure (EXAFS) studies [48, 55, 56] further support the occurrence of LMCT in Cu^2+^ frameworks coordinated by imidazole‐type ligands, reinforcing our structural assignment. Therefore, despite the limited precedent in literature, the combination of Cu L‐edge and N K‐edge spectroscopic data provides strong evidence for the formation of a nitrogen‐coordinated Cu‐MOF. Supporting this interpretation, Figure 5b presents the N K‐edge spectra of the Cu‐MOF, measured in TEY and FY. Both spectra display three distinct absorption features at 399.1 eV (A), 400.6 eV (B), and 401.6 eV (C), with similar line‐shapes confirming that the surface and bulk regions exhibit the same coordination chemistry. This profile closely resembles that of Zn‐ZIF‐8, indicating a comparable nitrogen coordination environment [57]. The low‐energy feature at 399.1 eV is assigned to the N 1s → π* transition of nitrogen atoms within the imidazolate linker that are directly coordinated to Cu(II) centers. Coordination to Cu lowers the transition energy due to LMCT and electron delocalization from the nitrogen lone pair into the partially filled Cu d orbitals. Similar low‐energy π* resonances have been reported in Cu‐imidazole and Zn–imidazole frameworks [57]. The second peak at 400.6 eV is attributed to noncoordinated or weakly interacting nitrogen atoms within the 2‐methylimidazolate linker, such as pyridinic sp^2^ N not directly bonded to Cu. This assignment is supported by reports of uncoordinated imidazole exhibiting π* transitions in the 400.5–401.0 eV range [58, 59]. The higher‐energy feature at approximately 401.6 eV may correspond to π* transitions of pyrrolic N (N–H), hydrogen‐bonded N sites (from coordinated H_2_O or partially decomposed MOF), or the onset of σ* transitions involving N─C or N─Cu bonds. These features typically appear at higher energies due to reduced electron density on nitrogen or greater localization, as observed in hydrogen‐bonded N species or imidazole molecules [58, 59].

In summary, the facilely synthesized Cu‐MOF features dual porosity with interconnected mesopores and macropores. Spectroscopic analyses confirm that the Cu^2+^ ions are coordinated to nitrogen atoms, forming a coordination environment characteristic of the ZIF‐8 framework. However, the morphology and crystalline properties of Cu‐MOF are distinct with respect to those of ZIF‐8. Recent studies have shown that Cu(II)‐based imidazolate frameworks can adopt flattened or distorted tetrahedral geometries, leading to alternative three‐dimensional network topologies [60]. Similarly, electron paramagnetic resonance (EPR) spectroscopy and DFT studies of Cu^2+^ substituted into ZIF‐8 confirm that while the local coordination remains tetrahedral, the CuN_4_ centers are distorted relative to ideal geometry [61]. These distortions can significantly affect the metal‐linker connectivity and favor alternative framework topologies. The flake‐like morphology of our Cu‐imidazolate material is consistent with previously reported growth mechanisms for Cu‐based imidazolate frameworks [33, 35, 36]. Prior studies show that Cu–N coordination tends to undergo slight tetrahedral distortion, which directs the assembly toward anisotropic, layer‐type growth rather than the isotropic sodalite (SOD) topology growth observed for Zn^2+^ and Co^2+^ [35]. Therefore, the observed differences in XRD pattern and flake‐like morphology are consistent with the formation of a distinct copper–imidazolate framework, one that preserves local tetrahedral Cu–N coordination but adopts a different global connectivity and growth habit from ZIF‐8 or ZIF‐67. These structural and chemical characteristics are expected to enhance the electrocatalytic performance of the material toward CO_2_RR.

### 2.2 Electrocatalytic Performance of Cu‐MOF for CO_2_RR

**FIGURE 6:**
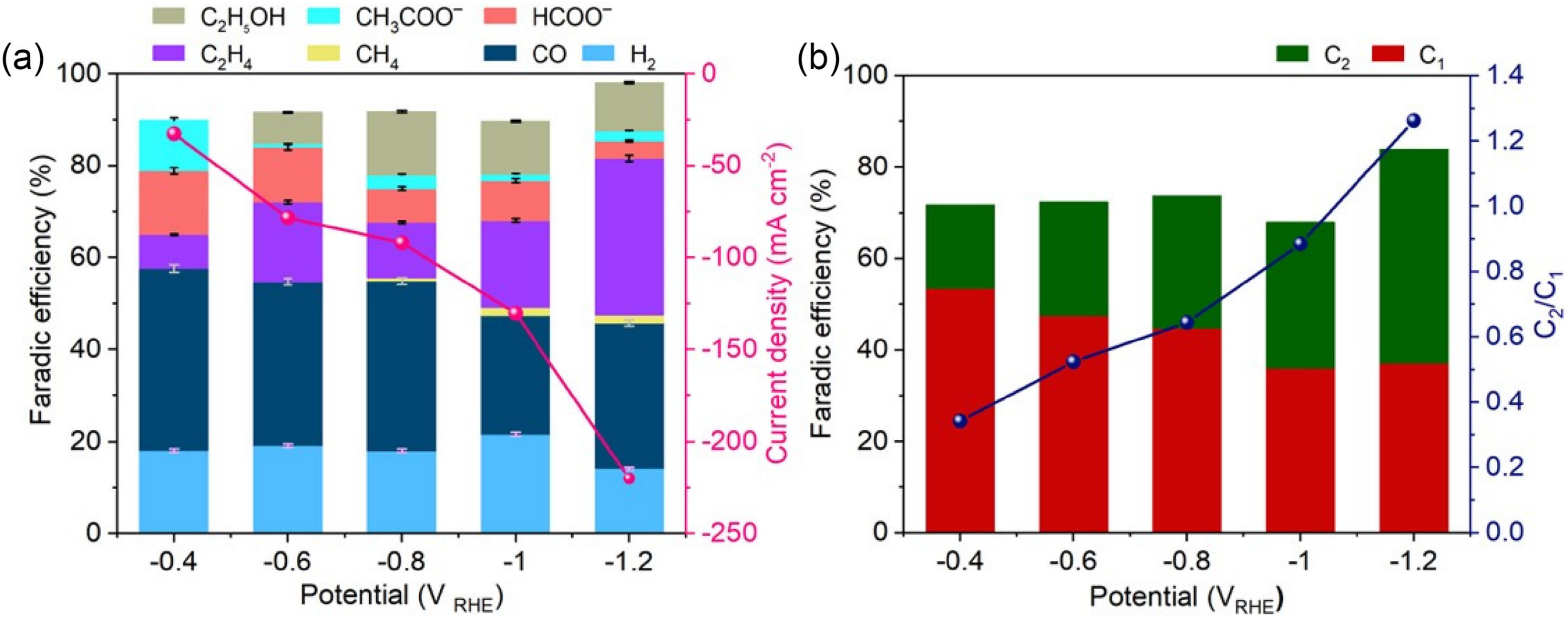
(a) FE of selectivity of CO_2_RR products and total current density of Cu‐MOF in a flow‐cell under various reduction potential in 1 M KOH catholyte and (b) FE and the ratio between productivity toward C_2_ and C_1_ products related to the data presented in (a).

The electrocatalytic activity of the Cu‐MOF toward CO_2_RR was first evaluated in a flow‐cell (schematic shown in Figure S4a), as presented in Figure 6a. By varying the applied potential in the range of –0.4 to –1.2 V_RHE_, the driving force for the reduction reactions was systematically tuned. Increasing the driving force enhances the reaction rate, yielding a maximum current density of 220 mA cm^–2^ at –1.2 V_RHE_. The maximum H_2_ production yield is limited to 20% (FE). CH_4_ and CH_3_COO^–^ are minor products. As overpotential increases, a fascinating trend emerges: the formation of C_1_ products, including CO and HCOO^–^ decreases, while the selectivity toward C_2_ products such as C_2_H_4_ and C_2_H_5_OH increases. At lower overpotentials from –0.4 to –0.8 V_RHE_, CO is the predominant product, with a nearly constant FE of around 37%. Upon further increasing overpotential, a significant shift in product selectivity occurs, accompanied by a decrease in CO production and an increase in C_2_H_4_ formation to a FE of 35%. In all the investigated potential range, while H_2_ production remains relatively constant, the ratio of C_2_/C_1_ products increased significantly, reaching a maximum value of 1.3 at –1.2 V_RHE_ with a total FE of 47% for C_2_ products (Figure 6b).

The influence of the catalyst loading was evaluated at different applied current densities from 100 to 400 mA cm^–2^. Figure S5 compares electrode loadings of 0.1, 0.25, 0.5, 1.0, 1.6, and 2.0 mg cm^–2^, showing no significant difference in electrode potential at identical current density, except at the highest current density tested. In contrast, catalyst loading has a pronounced effect on product distribution. Reducing the catalyst loading generally results in a substantial increase in C_1_ products accompanied by a decrease in C_2_ products. This loading‐dependent selectivity trend is consistent with previously reported observations for Cu nanoparticles [62]. Among the investigated loadings, 1.6 mg cm^−2^ exhibits the highest C_2_/C_1_ ratio, particularly at elevated current densities, and was therefore selected as the optimized catalyst loading for subsequent experiments.

**FIGURE 7:**
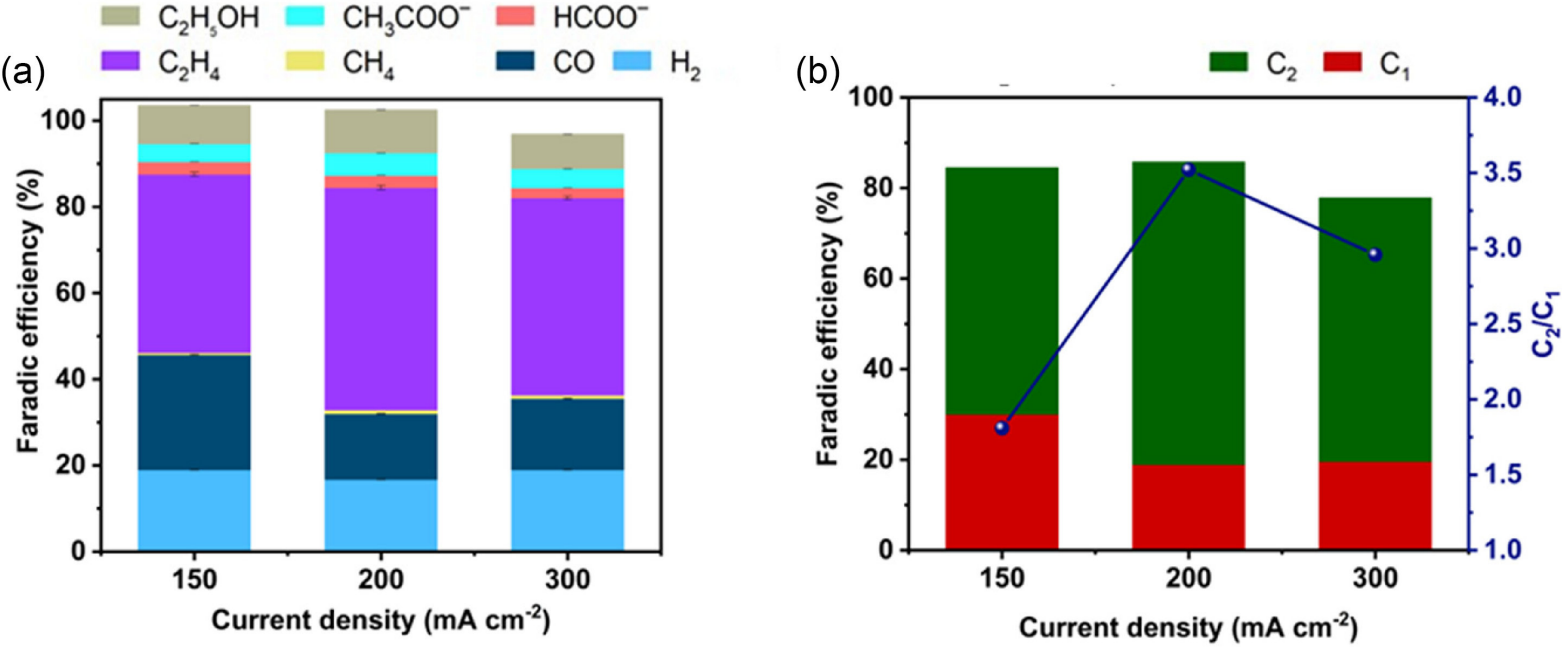
(a) FE of CO_2_RR products on Cu‐MOF GDEs in a zero‐gap cell at various current densities with 0.5 M KOH anolyte and (b) FE and the ratio between productivity toward C_2_ and C_1_ products related to the data presented in (a).

The Cu‐MOF GDEs were further evaluated in a zero‐gap electrochemical cell (EC‐cell; schematic shown in Figure S4b) at current densities of 150, 200 and 300 mA cm^–2^ with 0.5 M KOH anolyte. As shown in Figure 7a, the FE for H_2_ remains nearly constant at approximately 19% across all tested current densities, consistent with the behavior observed in flow‐cell configurations. In contrast, increasing the current density leads to a significant decrease in CO production and a remarkable increase in C_2_H_4_ selectivity. This trend culminates in a maximum FE of 51.6% for C_2_H_4_ at a current density of 200 mA cm^–2^. These results demonstrate that C_2_ products dominate the product distribution, accounting for 67% of the total products in the zero‐gap cell (Figure 7b). The CO_2_RR performance of the proposed Cu‐MOF is in line with the best performing catalysts reported in the literature, as summarized in Table S1. Notably, at comparable applied potentials and current densities, transitioning from a conventional liquid‐phase flow cell to a zero‐gap architecture shifts CO_2_RR selectivity away from CO and toward C_2_ products (especially C_2_H_4_). This behavior arises from fundamental differences in local CO_2_ availability, carbonate chemistry, water management, and intermediate retention between the two architectures [63, 64, 65, 66].

### 2.3 Stability Issues of CO_2_RR on Cu‐MOF Electrodes

**FIGURE 8:**
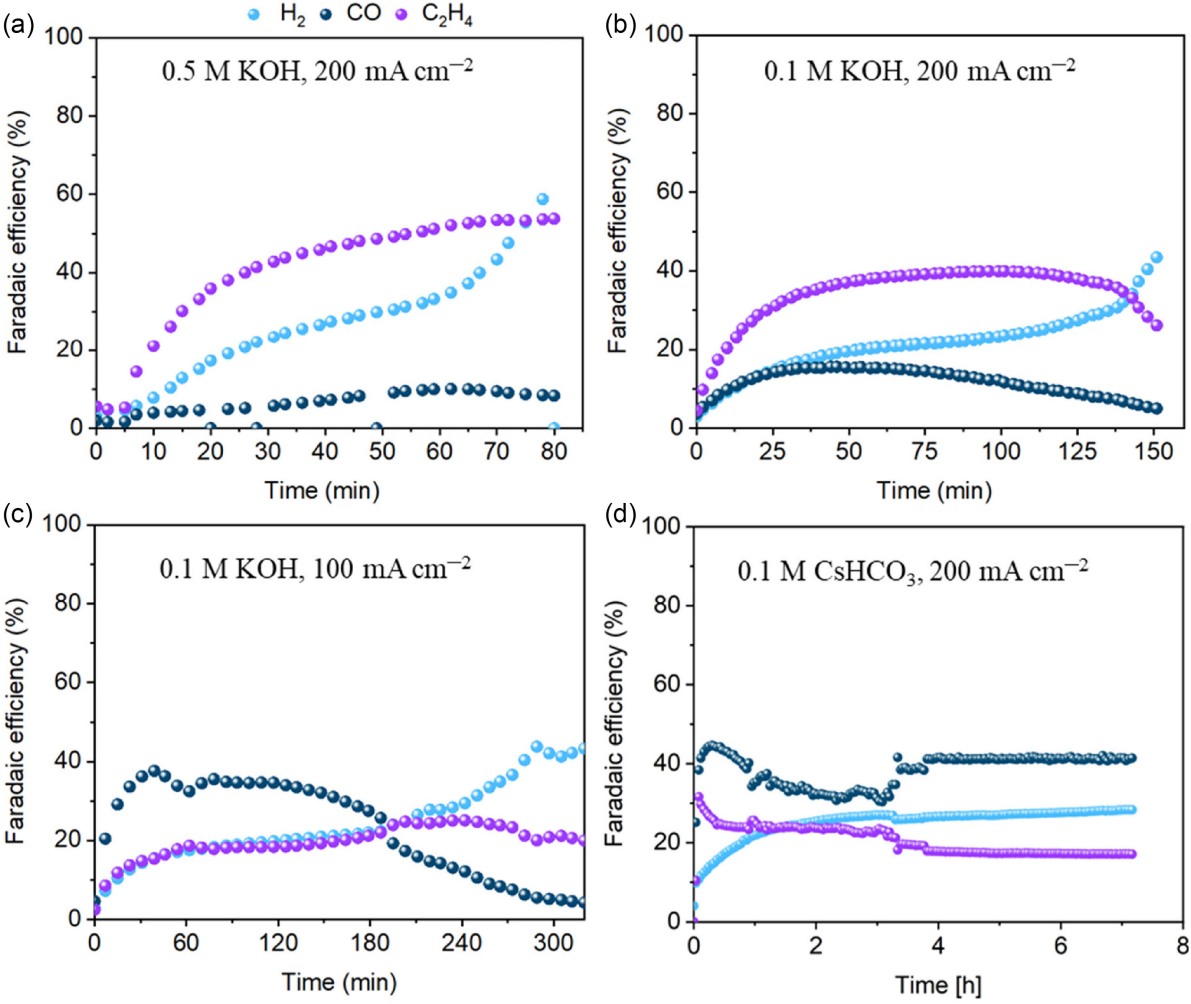
Time‐dependent CO_2_RR behavior of the Cu‐MOF electrode in the zero‐gap cell at different conditions: (a) 0.5 M KOH anolyte, 200 mA cm^–2^, (b) 0.1 M KOH anolyte, 200 mA cm^–2^, (c) 0.1 M KOH anolyte, 100 mA cm^–2^, and (d) 0.1 M CsHO_3_ anolyte, 200 mA cm^–2^ (quantification of liquid products within the first 1–2 h reveals formate (FE 5–10%), acetate (FE 2–6%), and ethanol (FE 5–10%) in all tests).

The time‐dependent CO_2_RR behavior of the Cu‐MOF electrodes in the zero‐gap cell is dependent on the anolyte composition and the current density. As shown in Figure 8a, with 0.5 M KOH anolyte, it reaches a high selectivity of ~51% for C_2_H_4_ after 1 h. Successively, a brutal increase in H_2_ formation is observed. Upon disassembly of the cell, substantial salt deposition was observed within the gas flow channels of the cathode flow field plate, corresponding to the backside of the GDE where CO_2_ enters (Figure S6). These salts, formed in situ from reactions of CO_2_ with OH^−^, block CO_2_ transport to the catalyst layer, creating localized CO_2_ starvation. Under these conditions, the hydrogen evolution reaction (HER) becomes kinetically favored, explaining the observed increase in H_2_ production over time. The stability of C_2_H_4_ production can be improved by lowering the anolyte concentration (Figure 8b), reducing the current density (Figure 8c), or using Cs^+^ instead of K^+^ cations (Figure 8d). These strategies help mitigate salt precipitation by decreasing cation diffusion to the cathode, limiting cation electromigration to the cathode, and increasing the solubility of formed salt at the cathode, respectively. When the salt deposition is largely mitigated, the eventual failure of the electrode is primarily attributed to flooding. During operation, the macroporous layer of the GDL is significantly affected by water accumulation driven by diffusion, which restricts CO_2_ mass transport [67]. In this study, replacing K^+^ with Cs^+^ led to a significant decrease in C_2_H_4_ selectivity. This observation contrasts with previous reports indicating that a decrease in the hydration radius of alkali metal cations enhances activity and selectivity toward C_2+_ products [67, 68, 69]. This discrepancy could be attributed to the specific catalyst properties, as the present work employs a MOF‐derived Cu catalyst, whereas the referenced studies predominantly used sputtered Cu electrodes.

**FIGURE 9:**
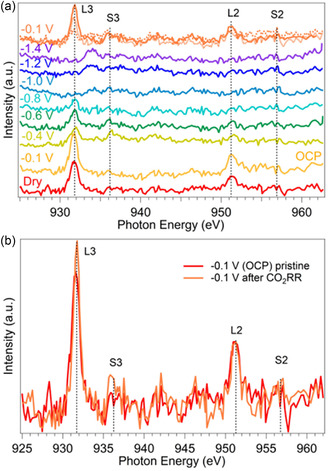
(a) Operando soft‐XAS experiment in EC‐cell for spray‐coated Cu‐MOF dried at 60°C: Cu L‐edge spectra recorded in FY mode for the dry electrode and their evolution from OCP to −1.4 V versus Ag/AgCl and (b) comparison of Cu L‐edge spectra of the Cu‐MOF at −0.1 V versus Ag/AgCl before and after CO_2_RR. A linear background was subtracted from all spectra.

Operando XAS measurements were performed to understand the changes in the electronic structure of Cu‐MOF under CO_2_RR conditions using a dedicated EC‐cell optimized for soft X‐ray experiments (Scheme Figure S7) [70]. The results demonstrate that Cu^2+^ atoms in the Cu‐MOF are progressively reduced to metallic Cu under CO_2_RR conditions, in agreement with the literature [71]. The catalyst ink was prepared following standard procedures by sonication in a hydroalcoholic Nafion solution and spray‐coated onto an Au‐coated (15 nm) SiN_*x*_ membrane (100 nm thickness), followed by drying at 60°C. The Cu L‐edge spectrum of the pristine Cu‐MOF film confirms the Cu^2+^ oxidation state in the as‐prepared electrode (Figure 9a). Compared to the powder sample (Figure 5a), the S3 satellite feature associated with LMCT from N to Cu is slightly shifted to lower photon energy, indicating a weaker ligand field and reduced Cu─N covalency bond withing the Cu‐MOF structure [72, 73]. This shift can be attributed to partial interaction with Nafion functional groups, as well as to the acidic and polar environment of the ionomer, which can modulate ligand‐field splitting without disrupting the overall MOF framework. Introduction of CO_2_‐saturated 0.1 M KHCO_3_ electrolyte in the EC‐cell does not modify the electronic structure of the catalyst and preserves the spectral intensity at the open circuit potential (OCP, −0.1 V vs. Ag/AgCl). Upon applying increasingly negative potentials up to –1.4 V versus Ag/AgCl, the Cu^2+^ signal progressively decreases, accompanied by the formation of reduced Cu species (Figure 9a). A fully quantitative determination of the Cu oxidation states is not feasible under the present operando conditions due to signal limitations; therefore, the analysis is based on relative changes in the Cu^2+^ spectral features. Specifically, the reduction proceeds gradually with decreasing potential: the intensity of the Cu^2+^ features decreases from ~100% at OCP to ~50% at −0.4 and −0.6 V, and further to ~30% at −0.8 V, indicating a progressive conversion of Cu^2+^ into lower oxidation states. However, at these intermediate potentials, the reduced species cannot be clearly detected or unambiguously assigned to either Cu^+^ or Cu^0^. This limitation arises from the significantly lower transition probabilities of Cu^+^ and Cu^0^ at the Cu L‐edge (approximately 7.5 and 13 times lower than that of Cu^2+^, respectively), resulting in weak spectral contributions close to the detection limit and partially masked by the background noise [74]. In addition, their spectral features overlap in the same photon energy range (see reference spectra in Figure 5a), preventing reliable deconvolution under the present experimental conditions. Finally, at more negative potentials (≤−1.2 V vs. Ag/AgCl), corresponding to CO_2_RR conditions, the Cu^2+^ features are fully suppressed and the spectra are dominated by contributions from reduced Cu species, consistent with the formation of metallic Cu, although the coexistence of Cu^+^ species cannot be excluded. Reversing the potential to −0.1 V versus Ag/AgCl, the sample fully restores the Cu^2+^ spectral features of the pristine electrode within 12 min (dashed curves at −0.1 V in Figure 9a). This reversible behavior suggests the formation of highly dispersed Cu species that can be readily re‐oxidized and recoordinated once the CO_2_RR potential is removed. Such dispersion is expected to promote a high density of accessible active sites and, consequently, a high *CO intermediate coverage, which favors C–C coupling reactions [75]. Comparison of Cu L‐edge spectra before and after CO_2_RR (Figure 9b) shows no significant changes in line shape or intensity, indicating that the electronic structure of the catalyst is preserved and that no substantial Cu dissolution occurs. The retention of Cu indicates that the local pH remains within a moderate range, as highly alkaline conditions would lead to Cu dissolution, as reported in our previous work [76]. This is consistent with the characteristics of the material, as mesopores enhance mass transport, particularly in the liquid phase [77], resulting in fast removal of OH^−^ generated in the reaction. This is consistent with the material’s characteristics, as mesopores enhance mass transport, particularly in the liquid phase [77], facilitating the rapid removal of OH^−^ generated during the reaction.

Overall, operando XAS reveals reversible changes in the electronic structure of Cu‐MOF, specifically the reduction to metallic Cu under CO_2_RR conditions and its recovery at OCP, occurring on a timescale of minutes. In contrast, salt precipitation is irreversible and accumulates overtime, causing a progressive loss of C_2_H_4_ selectivity and eventual cell failure over hours. Mitigating salt formation under optimized zero‐gap operating conditions could significantly enhance the long‐term stability of CO_2_RR on Cu‐MOF electrodes.

## 3 Conclusions

This work introduces a facile and green synthesis of a unique Cu‐MOF. The resulting material possesses a distinctive flake‐like morphology with dual porosity and a ZIF‐type framework defined by a Cu–N_4_ coordination environment. These structural and morphological features provide excellent active site accessibility and modulate reaction pathways, playing a pivotal role in enhancing the CO_2_RR performance. The Cu‐MOF electrode achieved a remarkable faradaic efficiency of 51.6% for ethylene and 67% for C_2_ products at an industrially relevant current density of 200 mA cm^−2^ in a zero‐gap cell. The combination of an energy‐efficient and sustainable synthesis route with high catalytic activity and selectivity presents a promising new avenue for designing advanced materials for CO_2_ conversion. Further optimization of the overall conversion process could significantly advance the practical implementation of CO_2_ electroreduction technologies.

## 4 Experimental Section

### 4.1 Materials

Copper (II) acetate tetrahydrate (Cu(OAc)_2_·4H_2_O, ≥98%, Sigma‐Aldrich) and 2‐methylimidazole (2‐MeIm, 99%, Sigma‐Aldrich) were employed as precursor materials for catalyst synthesis, with deionized water used as the solvent. Cesium hydrogencarbonate (CsHCO_3_, 99.9%, Sigma‐Aldrich), potassium hydroxide (KOH, ≥85%,), Nafion 117 solution (5 wt%, 40 μL), and isopropanol (IPA, ≥99.7%, Merck) were used in electrochemical testing. Ethanol was used as received without further purification.

### 4.2 Synthesis of Cu‐MOF

Cu‐MOFs were synthesized by modifying our previously established protocols, employing a steam‐assisted technique within a 100 mL Teflon‐lined stainless‐steel autoclave [78]. A mixture of Cu(OAc)_2_⋅4H_2_O (2.5 mmol) and 2‐MeIm (25 mmol) was introduced into a 30 mL quartz ceramic crucible. The crucible was positioned in the center of the autoclave, with 20 mL of DI water placed at the bottom to generate steam. The autoclave was sealed and heated at 120°C for 3 h. After cooling to room temperature, the resulting precipitate was collected by centrifugation at 4500 rpm and washed three times with a 2:1 (v/v) mixture of water and ethanol. The obtained powder was dried under vacuum at 60°C.

### 4.3 Preparation of Gas Diffusion Electrode

A certain amount of Cu‐MOF (7.5 mg) was dispersed in a mixture of isopropanol (450 μL) and Nafion 117 solution (40 μL, 5 wt%) through sonication for 30 min to create a homogeneous slurry. This slurry was then drop‐casted onto a 1 x 1.5 cm gas diffusion layer (SIGRACET GDL 28BC, Ion Power GmbH). The PTFE‐treated GDL with a microporous layer is widely used as a substrate for GDE fabrication, as it ensures efficient gas transport and helps mitigate rapid flooding [79]. The prepared GDEs were allowed to dry overnight at room temperature to ensure complete solvent evaporation. The resulting catalyst loading on the GDE was approximately 1.6 mg cm^–2^.

### 4.4 Physicochemical Characterization

XRD measurements were collected to verify the sample crystallinity. XRD data were acquired using a Rigaku Geigerflex D Max‐C Series diffractometer employing Cu‐Kα radiation, scanning in 0.02° 2*θ* increments, with a count time of 197 s per step.

The as‐prepared Cu‐MOF catalyst morphology was studied by FESEM (Supra40 from Carl Zeiss) at 5 kV. Additionally, elemental microanalysis of the as‐prepared Cu‐MOF catalyst was performed by SEM using a Hitachi SU8600 equipment operated at 10 kV, equipped with an energEDS detector (XFlash 7). The sample was mounted on carbon tape on an aluminum sample holder and coated with a thin Au/Pd layer prior to analysis.

The –196°C N_2_ adsorption–desorption isotherms were obtained using a Microtrac Belsorp MAX II. Before the experiment, the sample was degassed under vacuum at 150°C for 3 h using a heating rate of 5°C min^–1^. The specific surface area was determined by applying the BET theory to the –196°C N_2_ adsorption data (with an *R* ^2^ = 0.999), while the pore volume and pore size distribution curves were obtained by applying the NLDFT model.

TGA was performed using a Hitachi NEXTA STA 300 instrument under an air atmosphere between 25°C and 800°C and at a heating rate of 5°C min^–1^.

The actual Cu percentage in the Cu‐MOF samples was determined by ICP‐OES analysis on iCAP 7600 DUO spectrometer (Thermo Fisher Scientific). The ICP‐OES operating conditions for elemental quantification were as follows: auxiliary gas flow rate of 0.50 L min^–1^, cooling gas flow rate of 12 L min^–1^, nebulizer gas flow rate of 0.50 L min^–1^, nebulizer gas pressure of 180 kPa, RF power of 1150 W, and a peristaltic pump speed of 50 rpm. Elemental calibration was performed using a four‐point calibration curve (excluding the blank) at concentrations of 0.01, 0.1, 1, and 10 ppm.

Raman spectra were acquired in a Raman‐FT Bruker RFS/100S instrument equipped with Nd:YAG (1064 nm) laser at 100 mW and 4 cm^–1^ for 1000 scans.

FTIR spectroscopy was acquired using a Bruker Tensor 27 FTIR equipped with a SPECAC Golden Gate Diamond ATR accessory in the range of 4000–400 cm^–1^ and applying a resolution of 4  cm^–1^ and 256 scans.

Elemental analysis was made using a TruSpec 20 Micro 630‐200‐200 analyzer using 1–3 mg of sample. The combustion furnace was maintained at 1075°C, and the afterburner operated at 850°C. Carbon, hydrogen, and sulfur were detected via infrared absorption, while nitrogen was determined using thermal conductivity.

XPS analysis was performed via a PHI 5000 Versaprobe analyzer, by using an Al k‐alpha X‐ray monochromated source (1486.6 eV), with a double neutralizer charge system (Ar ions and electrons beams) to compensate surface charging phenomena. Survey and High Resolution (HR) scans have been collected choosing a Pass Energy (PE) equals to 187.85 and 23.50 eV respectively, a take‐off angle of 45°, and an X‐ray spot diameter of 100.0 μm. Binding energy scale was referred to C1s peak at 284.5 eV. MultiPak software (version 9.7.01) was used for data analysis.

Soft‐XAS characterization was performed at the BACH beamline of IOM‐CNR at the synchrotron radiation facility Elettra (Trieste, Italy). Measurements of the Cu L‐edge and N K‐edge and were performed in both TEY mode, by recording the drain current through the sample using a Keithley 428 current amplifier, and FY mode, using a multichannel plate (MCP) detector (Hamamatsu, F4655‐13) with an energy resolution better than 0.25 eV. The TEY and FY modes provide complementary information, probing the oxidation state of the Cu‐MOF at the surface and in the bulk, respectively.

Operando soft‐XAS experiments were conducted using a specific EC‐cell developed at BACH beamline (see scheme in Figure S7). Cu L‐edge XAS spectra were measured in FY mode, using a photodiode (IRD, AXUV100G) placed at 25° with respect to the incident X‐ray beam. A 0.1 M KHCO_3_ electrolyte solution, saturated with CO_2_, was flowed through the cell using a peristaltic pump (DG10‐BT103S). In‐operando XAS spectra were recorded at different applied potentials, from OCP to –1.4 V versus Ag/AgCl.

### 4.5 Electrochemical Characterization

The catalytic activity of Cu‐MOF for CO_2_RR was assessed using a customized three‐compartment flow‐cell and a commercial zero‐gap cell, as shown in Figure S4. In both cell configurations, a membrane was used to separate the anode and the cathode, which effectively mitigates the reoxidation of CO_2_RR products generated at the cathode. The working electrode consisted of a GDE loaded with Cu‐MOF.

In the flow‐cell, an iridium‐based anode (Ir‐MMO, ElectroCell) served as the counter electrode, and a mini‐Ag/AgCl electrode (1 mm, leak‐free LF‐1) was employed as the reference electrode. An anion exchange membrane (Sustainion 37–50, Dioxide materials) was positioned between the anolyte and catholyte. The 1 M KOH anolyte was recirculated, while the 1 M KOH catholyte was supplied in a single‐pass mode. Both the anolyte and catholyte flow rates were maintained at 4 mL min^–1^. A separate CO_2_ flow of 25 mL min^–1^ was injected into the gas compartment, facilitating diffusion through the GDE to reach the catalyst. Precise control of CO_2_ flow rates was maintained using Bruker mass flow controllers.

Zero‐gap cells are EC‐cells designed to minimize the distance between the electrodes and the membrane, effectively reducing ohmic losses and enhancing mass transport. An IrO_2_‐coated titanium mesh was used as the anode. An anion exchange membrane (Sustainion 37–50, Dioxide materials) was sandwiched between the cathode and the anode. The anolyte consisted of a 0.5 M KOH solution, which was continuously recirculated through the anode compartment using a peristaltic pump. The CO_2_ gas was directly fed into the cathode compartment. The continuous supply of CO_2_ ensures its availability for reduction at the catalyst surface.

Electrochemical measurements were performed using a CHI760D potentiostat. Each chronopotentiometry test, lasting at least 30 min, was conducted after applying 85% iR compensation to account for solution resistance. Each measurement was repeated at least twice. Measured electrode potentials were rescaled by applying the Nernst equation, and the reported values are referred to the RHE [78].

Gaseous products generated during CO_2_RR experiments were analyzed in real time using a micro gas chromatograph (µGC, Fusion, INFICON). This µGC system features two separate channels for enhanced separation and detection: Channel 1: A 10 m Rt‐Molsieve 5 Å column is employed to separate and detect small and permanent gases, such as H_2_, CO, and CH_4_; Channel 2: An 8 m Rt‐Q‐Bond column is used for the separation and detection of larger molecules, such as CO_2_, C_2_H_6_, and C_2_H_4_. Each channel is equipped with a sensitive microthermal conductivity detector (µTCD) for accurate quantification of the separated gas components. The µGC inlet is directly connected to the cathodic side of the EC‐cell, allowing continuous monitoring of the gas‐phase products formed during CO_2_RR. Liquid products from the CO_2_RR experiments were analyzed using high‐performance liquid chromatograph (Nexera Series, Shimadzu HPLC). The HPLC was equipped with a photo diode array detector, a refractive index detector, and a ReproGel H^+^ (Dr Maisch 300  ×  8 mm) column. A solution of 9.0 mM H_2_SO_4_ (flow rate of 1.0 mL min^−1^) was used as the mobile phase. This acidic mobile phase helps to improve peak resolution and sensitivity for the target analytes. The efficiency of the CO_2_RR process was evaluated by calculating the FE for each detected product. It is determined by Equation (1)

(1) $$FE=\frac{nNF}{Q}$$

In this equation: *n* represents the stoichiometric electron requirement per molecule of the target product (e.g., 2 electrons for CO, HCOO^−^, HCOOH, and H_2_; 12 electrons for C_2_H_4_ and C_2_H_5_OH; 8 electrons for CH_4_, CH_3_COO^−^, and CH_3_COOH). *N* denotes the measured number of moles of the product formed. F is Faraday’s constant. *Q* is the total electrical charge passed during the electrolysis process. The FE represents the percentage of the total charge consumed during electrolysis that is used to produce a specific product. By combining the results of gas‐phase and liquid‐phase product analyses, a comprehensive understanding of the CO_2_RR process and the catalyst’s performance can be gained.

## Author Contributions

**Juqin Zeng** suggested and supervised the project. **Mitra Bagheri**, **Mirtha A. O. Lourenço**, **Micaela Castellino**, **Silvia Nappini** **,** **Felicia Di Costola**, **Julien K. Dangbegnon**, and **Elena Magnano** performed the measurements and analyzed the data. **Fabrizio Pirri** **,** **Luís Mafra,** **Mirtha A. O. Lourenço**, and **Adriano Sacco** provided resources. **Mitra Bagheri**, **Mirtha A. O. Lourenço**, and **Juqin Zeng** wrote the original manuscript. All authors reviewed and edited the article. All authors contributed to the discussions and commented on the article.

## Funding

This study was supported by Ministero dell’Università e della Ricerca (Project code: IR0000027, CUP: B33C22000710006, Project title, Project No. 2022A2A9NW‐CUP: B53D2301357000), HORIZON EUROPE European Research Council (Grant Agreement 865974, grant agreement No. 101090287), and Fundação para a Ciência e a Tecnologia (COMPETE2030‐FEDER‐00776600).

## Conflicts of Interest

The authors declare no conflicts of interest.

## Supporting information

Supplementary Material

## Data Availability

The data that support the findings of this study are available from the corresponding author upon reasonable request.
