# Supplementary material for: Mesoporous Copper‐Based Metal‐Organic Framework Flakes as a Promising Platform for Electrosynthesis of Ethylene from Carbon Dioxide
Source: Small Sci. 2026 Jun 11;6(6):e70323. doi: 10.1002/smsc.70323 (PMC13274310; doi:10.1002/smsc.70323)
Supplement: Supplementary file 1 — Supplementary Material [file SMSC-6-e70323-s001.pdf]

Supporting information

Mesoporous Cu-MOF flakes as a promising platform for electrosynthesis of ethylene from carbon dioxide

Mitra Bagheri[1,2], Mirtha A. O. Lourenço[3], Micaela Castellino[1,2], Silvia Nappini[4], Felicia Di Costola[1,2], Julien K. Dangbegnon[1], Elena Magnano[4], Luís Mafra[3], Fabrizio Pirri[1,2], Adriano Sacco[1], Juqin Zeng[1,2*]*

[1]Istituto Italiano di Tecnologia – IIT, Center for Sustainable Future Technologies (CSFT), Via Livorno 60, Turin 10144, Italy

[2]Department of Applied Science and Technology (DISAT), Politecnico di Torino, Corso Duca degli Abruzzi 24, Turin 10129, Italy

[3]CICECO – Aveiro Institute of Materials, Department of Chemistry, University of Aveiro, 3810-193, Aveiro, Portugal

[4]CNR - Istituto Officina dei Materiali (IOM), Trieste, Basovizza 34149, Italy

*Corresponding authors: mitra.bagheri@iit.it; juqin.zeng@iit.it

The Cu content in the Cu-MOF was initially quantified by thermogravimetric analysis (TGA) up to 800 °C under an air atmosphere (Figure S 1). The TGA curve displayed a multi-step weight loss pattern, with the material remaining thermally stable up to approximately 225 °C. An initial weight loss of about 18.0% occurred beyond this temperature and is attributed to the evaporation of solvents (H_2O and EtOH) and the residual organic linker (2-methylimidazole, boiling point 267–268 °C. At higher temperatures, around 400 °C, further weight loss was observed, corresponding to the decomposition of the MOF framework and the release of volatile components. After complete decomposition, the residual weight was approximately 35.5%, attributed to the formation of inorganic copper oxide species in air corresponding to an estimated Cu content of ~28.4 wt.%.

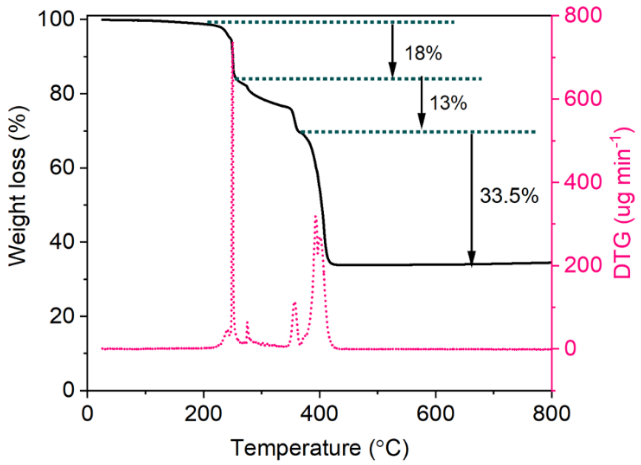

Figure S 1. TGA curves of Cu-MOF.

Both Raman and FTIR are vibrational spectroscopic techniques used to study the chemical composition and structure of materials.

Figure S 2 presents a comparative analysis of the FTIR spectra of 2-methylimidazole and the synthesized Cu-MOF, revealing a significant spectral shift and the emergence of new bands, indicative of metal-ligand coordination. The O-H stretching vibration band around 3100-3400 cm^{-1} region was observed in both spectra, attributed to adsorbed water molecules with different degrees of H-bondings. C-H stretching vibrations of the methyl and aromatic ring groups appear in the 2900-3000 cm^{-1} region for both compounds [1]. Changes in peak width and shape can provide insights into structural order. The sharper bands in the Cu-MOF spectrum suggest increased order, likely due to ligand rigidification upon metal coordination, which reduces vibrational freedom [2,3]. In the 1600-1500 cm^{-1} region, intense bands characteristic of C=C and C=N stretching vibrations of the aromatic ring are observed in both spectra [1]. Shifts in peak intensity and position, such as noticeable shift to higher wavenumbers in the Cu-MOF indicate stronger C=N bond resulting from coordination of the imidazole nitrogen atoms to the Cu (II) ions [1,4]. In the 1400-1300 cm^{-1} region, C-H bending vibrations are observed, while C-N stretching vibrations are present in the 1100-1000 cm^{-1} range. A new, sharp band emerged at 433 cm^{-1} in the Cu-MOF spectrum, characteristic of metal-ligand (Cu-N) stretch of 2-methylimidazole reveals characteristic peaks of its functional groups. A broad band in the 2300-2200 cm^{-1} region is indicative of hydrogen bonding involving the N-H group [1], while a sharp peak around 1600 cm^{-1} confirms the presence of the N-H bending vibrations in the imidazole ring [1]. The disappearance or reduction of the peak at 1851 cm^{-1}, attributed to the resonance between N-H stretching and N-H...N out-of-plane bending, confirms the involvement of the N-H group in metal coordination [1]. Additional spectral peaks shifts, and broadening suggest alterations in the electronic environment of the ligand upon metal binding.

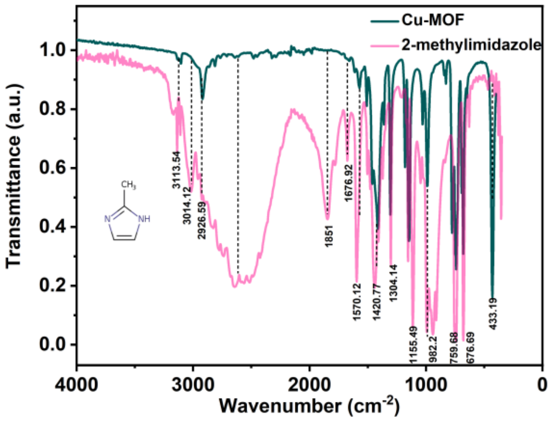

Figure S 2. FTIR spectra of Cu-MOF and 2-methylimidazole.

The Raman spectrum of the synthesized Cu-MOF confirms its successful formation, featuring sharp and intense peaks at 690, 1023, 1183, 1462, and 2920 cm^{-1} (Figure S 3). These peaks correspond to various vibration modes of the 2-methylimidazole aromatic ring: C=C stretching (690 cm^{-1}), C-H bending (1023 cm^{-1}), C-N stretching (1183 cm^{-1}) within the imidazole rings, methyl bending (1462 cm^{-1}), C-H stretching of the methyl groups (2920 cm^{-1}) [1]. The vibrational modes associated with the Cu-N bonds within the metal-organic framework typically appear in the lower frequency region of the Raman spectrum. The peak observed at 293 cm^{-1} can be attributed to the Cu-N bonds [5], although these modes are often obscured by overlapping vibrational, making precise identification challenging.

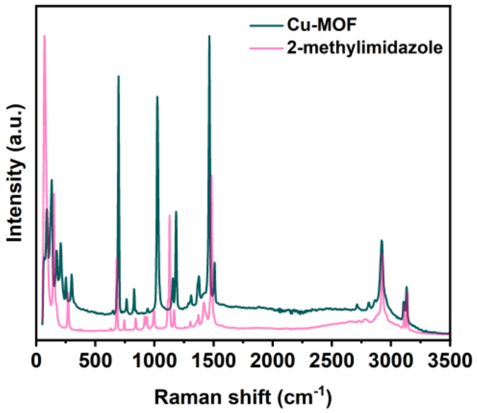

Figure S 3. Raman spectrum of Cu-MOF and 2-methylimidazolem

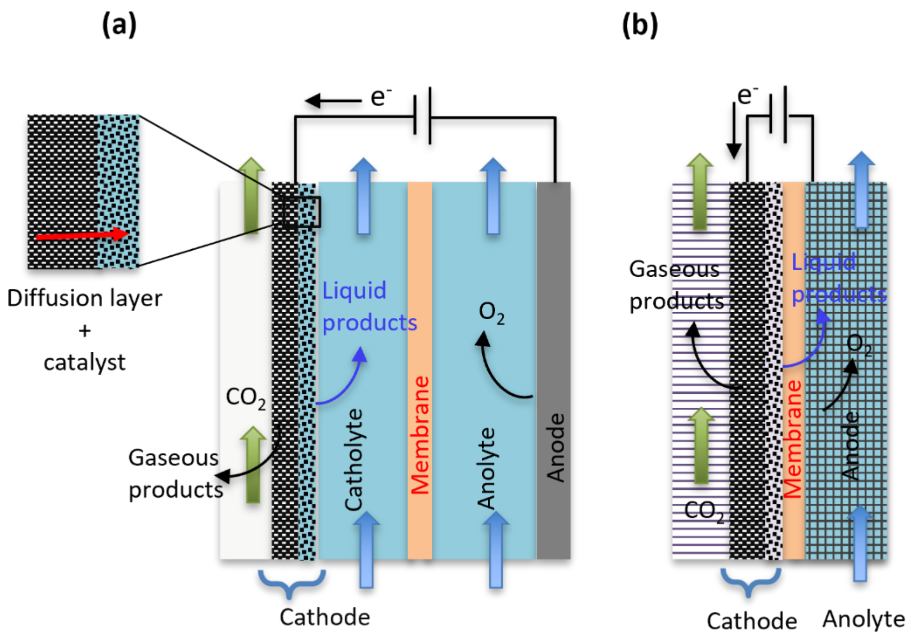

Figure S 4. Schematic diagrams of CO_2 electrolyzers: (a) flow-cell and (b) MEA-cell

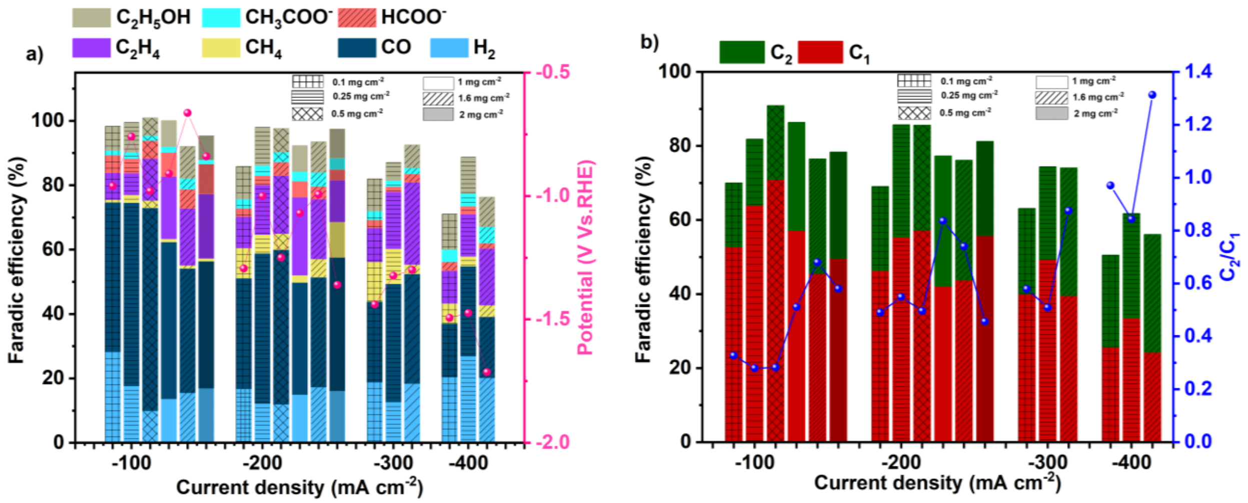

Figure S 5. (a) Comparison of (FE) of CO_2RR products for Cu-MOF with different loadings (0.1, 0.25, 0.5, 1.0, 1.6 and 2.0 mg cm^{-2}) in a flow cell under various applied current densities in 1 M KOH, including the corresponding reach potentials, (b) FE and the ratio between productivity towards C_2 and C_1 products related to the data presented in part (a).

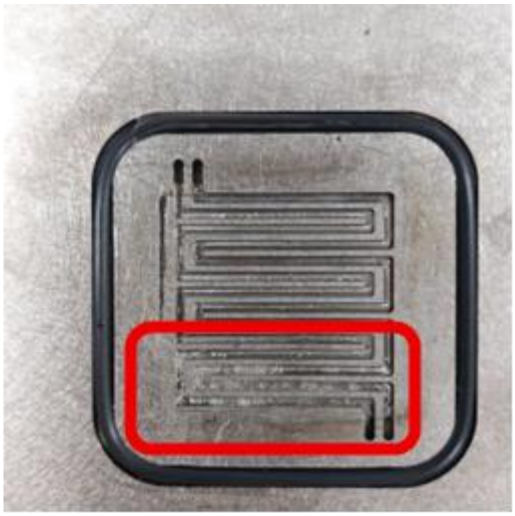

Figure S 6. Salt deposition on the flow channels.

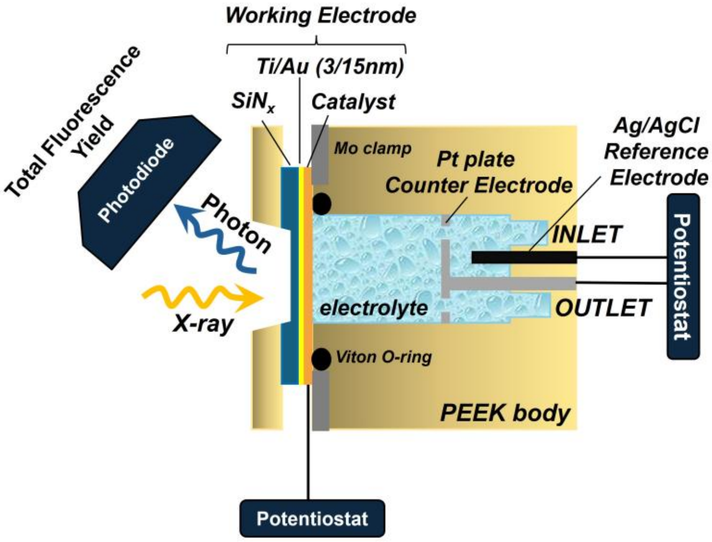

Figure S 7. Scheme of the EC-cell and measurement setup for operando soft-XAS experiments implemented at the BACH beamline, Elettra Sincrotrone Trieste, reproduced from: Napal Azcona, I. X-Ray Spectroscopies for Hydrogen Based Economy (Doctoral Dissertation).

Table S1. Comparison of representative Cu-based catalysts for CO_2 electroreduction toward C_2 products, including major products, Faradaic efficiency (F including major products, Faradaic efficiency (FE), C_2/C_1 ratio, reaction conditions, and key features.

Catalyst class	Catalyst system	Major product(s)	FE (%)	C_2/C_1 ratio	Potential / Cell voltage vs. RHE (V)	Current density ($mA \cdot cm^{-2}$)	Electrolyte	Ref.
**Cu-based with different functional groups (−NH_2, −COOH, G−, N−, and −OH)**	Cu_1Ni_2@N-MWCNT and Cu_1Ni_2@MWCNT-OH	C_1	90	—	-0.53		0.5 M $KHCO_3$	[6]
**Bimetallic / Alloy Cu**	CuSn bimetallic	CO	94	—	-1.1	7.9	0.1 M $KHCO_3$	[7–9]
		$HCOO^-$	92		-0.95	9.01		
		CO	85		-1.1	11.5		
	CuSb bimetallic	CO	96	—	-1.2	74	0.1 M $KHCO_3$	[10,11]
		CO	97		-0.6	42.8	0.1 M $KHCO_3$	
	Cu–(6%Ag) alloy film	C_2H_4, EtOH	~60 25	—	-0.7	-300		[12]
	Cu–Al alloy	C_2H_4	~75	—	-1.8	400	1 M KOH	[13]
	Cu–Zn alloy	C_2H_4, EtOH	~40–25	—	-0.8	-93		[14]
**MOF-Derived Cu**	Cu@Cu_xO core–shell	C_2H_4	49.8	—	-1.58	150		[15]
	Cu_2O@Cu-MOF	CH_4	63	—	-1.7	—	0.1 M $KHCO_3$	[16]
	Cu(111)@MOF	C_2H_4	44.2	—	-1.2	8.2		[17]
	CE-modified CuBTC	C_2H_4	52		-2	120	1 M KOH	[18]
	Cu-MOF (Cu-BDC)	C_2H_4	26.12		-1.36	-35	0.1 M $KHCO_3$	[19]
**Pristine Cu-MOFs**	Cu-DBC MOF	CH_4	56	—				[20]
	Cu-HHTP MOF	CO	42	—	-1.4	11.4	0.1 M $KHCO_3$	
	Cu-THQ MOF	CO	91	—				
	Cu-MOF-20	EtOH	82.5		-1	8.7		[21]
	Cu(I)-BTC	C_2H_4	57		-1.6	-40 to -60	0.1 M $KHCO_3$	[22]
**This Work**	**Proposed Cu-MOF**	**C_2H_4**	**51.6**	**3.5**	**-1.2**	**200**	**1 M KOH**	**—**

[1] R. Majidi, A. Farhadi, I. Danaee, N. B. Panah, D. Zarei, S. Nikmanesh, *Prog. Org. Coat.* **2023**, *183*, DOI 10.1016/j.porgcoat.2023.107803.

[2] X.-L. Lv, S. Yuan, L.-H. Xie, H. F. Darke, Y. Chen, T. He, C. Dong, B. Wang, Y.-Z. Zhang, J.-R. Li, H.-C. Zhou, *J. Am. Chem. Soc.* **2019**, *141*, 10283.

[3] B. C. Smith, *Spectroscopy* **2024**, 31.

[4] A. M. Kale, R. Manikandan, C. Justin Raj, A. Dennyson Savariraj, C. Voz, B. C. Kim, *Mater. Today Energy* **2021**, *21*, 100736.

[5] S. Duan, L. Wu, J. Li, Y. Huang, X. Tan, T. Wen, T. Hayat, A. Alsaedi, X. Wang, *J. Hazard. Mater.* **2019**, *373*, 580.

[6] M. Wang, Z. Cai, B. Zhang, K. Yang, T. Shou, M. T. Bernards, P. Xie, Y. He, Y. Shi, *Energy & Fuels* **2022**, *36*, 5833.

[7] W. Ju, J. Zeng, K. Bejtka, H. Ma, D. Rentsch, M. Castellino, A. Sacco, C. F. Pirri, C. Battaglia, *ACS Appl. Energy Mater.* **2019**, *2*, 867.

[8] X. Jiang, X. Wang, Z. Liu, Q. Wang, X. Xiao, H. Pan, M. Li, J. Wang, Y. Shao, Z. Peng, Y. Shen, M. Wang, *Appl. Catal. B* **2019**, *259*, 118040.

[9] J. Zeng, K. Bejtka, W. Ju, M. Castellino, A. Chiodoni, A. Sacco, M. A. Farkhondehfal, S. Hernández, D. Rentsch, C. Battaglia, C. F. Pirri, *Appl. Catal. B* **2018**, *236*, 475.

[10] H. Huang, K. Yue, K. Lei, B. Y. Xia, Y. Yan, *The Journal of Physical Chemistry C* **2024**, *128*, 12067.

[11] J. Zeng, M. R. Fiorentin, M. Fontana, M. Castellino, F. Risplendi, A. Sacco, G. Cicero, M. A. Farkhondehfal, F. Drago, C. F. Pirri, *Appl. Catal. B* **2022**, *306*, 121089.

[12] T. T. H. Hoang, S. Verma, S. Ma, T. T. Fister, J. Timoshenko, A. I. Frenkel, P. J. A. Kenis, A. A. Gewirth, *J. Am. Chem. Soc.* **2018**, *140*, 5791.

[13] M. Zhong, K. Tran, Y. Min, C. Wang, Z. Wang, C.-T. Dinh, P. De Luna, Z. Yu, A. S. Rasouli, P. Brodersen, S. Sun, O. Voznyy, C.-S. Tan, M. Askerka, F. Che, M. Liu, A. Seifitokaldani, Y. Pang, S.-C. Lo, A. Ip, Z. Ulissi, E. H. Sargent, *Nature* **2020**, *581*, 178.

[14] Y. Baek, H. Song, D. Hong, S. Wang, S. Lee, Y.-C. Joo, G.-D. Lee, J. Oh, *J. Mater. Chem. A Mater.* **2022**, *10*, 9393.

[15] K. Yao, Y. Xia, J. Li, N. Wang, J. Han, C. Gao, M. Han, G. Shen, Y. Liu, A. Seifitokaldani, X. Sun, H. Liang, *J. Mater. Chem. A Mater.* **2020**, *8*, 11117.

[16] X. Tan, C. Yu, C. Zhao, H. Huang, X. Yao, X. Han, W. Guo, S. Cui, H. Huang, J. Qiu, *ACS Appl. Mater. Interfaces* **2019**, *11*, 9904.

[17] Z.-H. Zhao, K. Zheng, N.-Y. Huang, H.-L. Zhu, J.-R. Huang, P.-Q. Liao, X.-M. Chen, *Chemical Communications* **2021**, *57*, 12764.

[18] X. Zheng, S. Yang, D. Chen, Y. Kong, T. Cui, X. Zheng, H. Fu, W. Xue, S. Li, C. Cheng, H. Chen, R. Li, J. Xu, *Chemical Communications* **2025**, *61*, 2993.

[19] Z. Ni, T. Yu, X. Li, J. Li, C. Liu, X. Liu, R. Guo, *Int. J. Hydrogen Energy* **2025**, *138*, 415.

[20] Y.-Y. Liu, H.-L. Zhu, Z.-H. Zhao, N.-Y. Huang, P.-Q. Liao, X.-M. Chen, *ACS Catal.* **2022**, *12*, 2749.

[21] B. Shao, D. Huang, R. Huang, X. He, Y. Luo, Y. Xiang, L. Jiang, M. Dong, S. Li, Z. Zhang, J. Huang, *Angew. Chem. Int. Ed.* **2024**, *63*, DOI 10.1002/anie.202409270.

[22] J. Deng, L. Qiu, M. Xin, W. He, W. Zhao, J. Dong, G. Xu, *Small* **2024**, *20*, DOI 10.1002/smll.202311060.
